# Addressing Cancer Screening Inequities by Promoting Cancer Prevention Knowledge, Awareness, Self-Efficacy, and Screening Uptake Among Low-Income and Illiterate Immigrant Women in France

**DOI:** 10.3389/ijph.2021.1604055

**Published:** 2021-06-15

**Authors:** Maria De Jesus, Christelle M. Rodrigue, Sarah Rahmani, Christian Balamou

**Affiliations:** ^1^ Collegium de Lyon, Université de Lyon, Lyon, France; ^2^ School of International Service, American University, Washington, DC, United States; ^3^ Center on Health, Risk, and Society, American University, Washington, DC, United States; ^4^ Centre Régional de Coordination des Dépistages des Cancers Auvergne-Rhône-Alpes (CRCDC AuRA), Site de l’Ain Bourg-en-Bresse, France

**Keywords:** cancer inequities, cancer screenings, cancer prevention, cancer knowledge and awareness, immigrant, low literacy, low income, France

## Abstract

**Objective:** Cancer screening rates are suboptimal for disadvantaged populations in France, yet little evidence exists on their cancer-related knowledge and screening barriers. The main objective of this study was to examine cancer-related knowledge, awareness, self-efficacy, and perceptions of screening barriers among low-income, illiterate immigrant women in France following an 8-weeks cancer educational intervention.

**Methods:** Semi-structured qualitative interviews were conducted with 164 female participants in the Ain department of France between January 2019 and March 2020. Adopting the Health Belief Model as an intervention and analytic framework, salient themes were identified using qualitative thematic analysis.

**Results:** Increased levels of perceived susceptibility to and perceived severity of cancer contributed to higher motivation to get screened. Barriers to screening included low French proficiency, shame surrounding illiteracy, and constant worries due to precarious living conditions. Perceived benefits (e.g., valuing one’s health and health-promoting behaviors), cues to action from a trusted source, and greater self-efficacy (e.g., more autonomous in healthcare-seeking) outweighed perceived barriers, including cultural barriers.

**Conclusions:** Implications include developing audience-responsive targeted cancer screening communication strategies and educational materials to increase screening rates and reduce cancer and cancer screening inequities.

## Introduction

Cancer is the second leading cause of death globally with an estimated 9.6 million deaths in 2018 [1]. Evidence demonstrates inequities in cancer incidence, survival, and mortality between high-income and low-to-middle income countries, as well as between groups living within the same country [1, 2]. Social inequities impact an individual’s exposure to risk factors and the likelihood of developing cancer, and their access to screening, diagnostic, and treatment facilities, and whether they have access to palliative care [1, 2].

In France, in 2018, the number of new cases of cancer was estimated at approximately 382,000 and the number of deaths at approximately 157,400, with cancer as the leading cause of mortality for all sexes combined [3]. Breast cancer is considered to be the most common cancer in France and the most frequent fatal cancer for women [4]. There are 58,000 new cases per year, with one woman in eight affected by cancer [4]. Following breast cancer, colorectal cancer is the second most common cancer in women, and the third most common cancer in men [4]. It is the second most deadly cancer in France [3]. The colorectal cancer incidence in metropolitan France in 2018 was 43,336 new cases and 17,117 deaths [3]. Cervical cancer affects far fewer women than breast or colorectal cancer, but it is the second most common cancer in women between 30 and 45 years of age [4]. In 2018, the number of new cases of cervical cancer was 2,920 and the number of deaths was estimated at 1,117 in France [5].

Organized cancer screening programmes have been strongly promoted in France and other European countries since the 1980s. These programmes were progressively implemented in France in 2003 following a recommendation of the Council of the European Union and the publication of recommendations for quality screening [6]. Screening programmes are organized at the regional level by Regional Cancer Screening Coordination Centers (Centres Régionaux de Coordination des Dépistage des Cancers or CRCDCs).

The third National Plan Against Cancer 2014–2019 in France prioritized the reduction of inequities in access to cancer screenings. One of the objectives was “to combat inequality in uptake of and access to screening, and to increase the efficiency of programmes, in order to reduce avoidable deaths and the more severe treatments associated with delayed care” [7]. The French Cancer Registry confirms a positive impact of the screening campaigns on reducing the mortality rate among participants, although the participation rates are still lower than expected [8–10]. The participation rate in France for organized breast cancer screening was 48.6% in 2019 [11], colorectal cancer screening was 30.5% in 2018–2019 [12], and cervical cancer was 59.5% in 2019, a rate which has been stable since 2012 [13].

Low participation rates across the different types of cancer screening are closely related to lower levels of socioeconomic status and education [14–17]. Moreover, ethnic minority, migrant, and immigrant populations have lower participation screening rates compared to non-ethnic and native-born populations [18–20]. Language and inadequate health literacy skills are cited as main barriers to accessing information and screening among these populations [19–22]. To reduce these inequities, audience-centered approaches are needed with relevant activities offered, especially for members of low-income areas who are most affected by cancer and cancer screening inequities [23].

**FIGURE 1:**
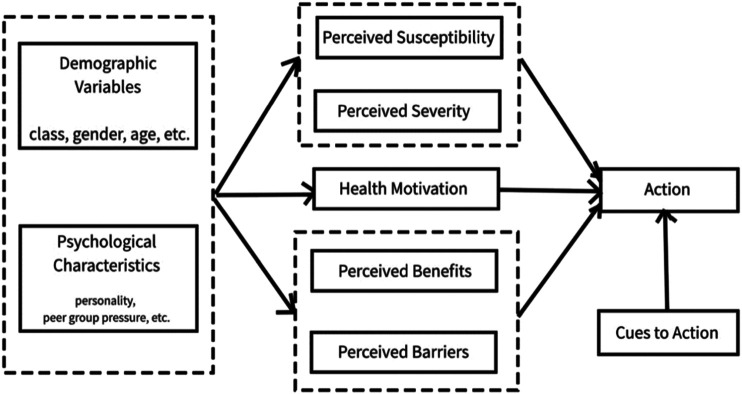
The Health Belief Model Action to Promote Organized Cancer Screenings for the Public Developing Literacy in the Ain” (Action de promotion du Dépistage Organisé des Cancers auprès des Publics en cours d'alphabétisation dans l'Ain (ADOCPA), France, 2019–2020. “Action to Promote Organized Cancer Screenings for Populations Developing Literacy in Ain” (Action de promotion du Dépistage Organisé des Cancers auprès des Publics en cours d’alphabétisation dans l’Ain (ADOCPA), France, 2019–2020.

Given that cancer screening rates are suboptimal for low-income, low-literate, and other disadvantaged populations in France [8–10], evidence is needed to develop responsive interventions that increase cancer screening uptake and reduce cancer inequities. Very little is known on the cancer-related knowledge, awareness, self-efficacy, and perceptions of screening barriers among low-income, illiterate immigrant women in France. To fill this gap in the literature, the main objective of this study was to examine in-depth the cancer-related knowledge, awareness, self-efficacy, and perceptions of screening barriers of this understudied population following an 8-weeks cancer educational intervention. The Health Belief Model (HBM) [24] as applied to cancer prevention was used to guide the intervention and data analysis (Figure 1). The HBM has been widely used to measure the health beliefs and behaviors about cancer screening [25–27].

## Methods

The current study was part of a larger intervention study titled “Action to Promote Organized Cancer Screenings for the Public Developing Literacy in the Ain” [Action de promotion du Dépistage Organisé des Cancers auprès des Publics en cours d'Alphabétisation dans l'Ain (ADOCPA)]. ADOCPA has two main objectives: [1]: to increase the participation rate of organized screening for colorectal, breast and cervical cancer among illiterate populations through health education sessions followed by delivery of a Faecal Immunochemical test (FIT) screening kit for colorectal cancer or by invitation letters to get screened; [2]; to increase illiterate individuals’ self-efficacy in obtaining cancer screenings. The educational intervention approach and materials were tailored for a population with very low literacy levels. For example, we used short, common words as well as images to describe different cancers, and we color coded each cancer screening to increase participants’ comprehension of each screening. Sentences were written at a first-grade level and were kept short, approximately 10 words in length and in the active voice. We also used easily legible font types and sizes. Finally, the educational sessions were interactive, whereby we encouraged the active participation of all the women in each session.

### Setting, Intervention Design, and Recruitment

The Auvergne-Rhône-Alpes Regional Cancer Screening Coordination Center (Centre Régional de Coordination des Dépistages des Cancers Auvergne-Rhône-Alpes or CRCDC AuRA) is mandated by the Ministry of Health for the Auvergne-Rhône-Alpes region in France to develop and implement screening programmes for breast, colorectal and cervical cancers. These three cancer screenings were targeted as part of a larger educational intervention promoting organized cancer screening programs in different regions in France. The Auvergne-Rhône-Alpes region is located in southeast-central France. The region is the third largest area in metropolitan France, and has a population of 7,994,459 [28]. It consists of 12 departments and one territorial subdivision. The city of Lyon is the administrative centre of the region.

**FIGURE 2:**
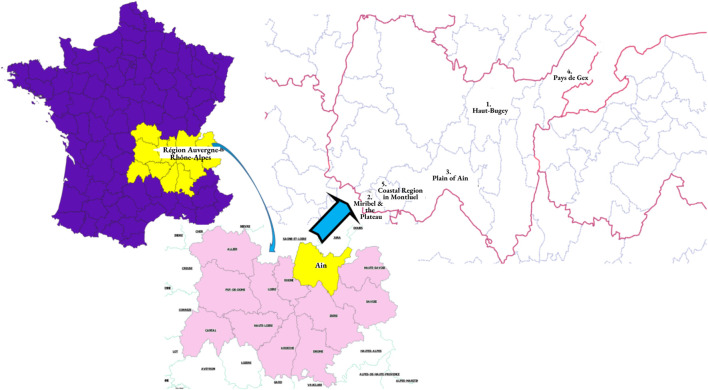
Maps depicting study sample setting: 1) Auvergne-Rhône-Alpes region in France; 2) Department of Ain; 3) Five study sample territories of Ain. “Action to Promote Organized Cancer Screenings for Populations Developing Literacy in Ain” (Action de promotion du Dépistage Organisé des Cancers auprès des Publics en cours d’alphabétisation dans l’Ain (ADOCPA), France, 2019–2020.

Given that Ain is a department in the Auvergne-Rhône-Alpes region (Figure 2) with low cancer screening participation rates, a cancer education and prevention intervention to promote cancer-related knowledge, awareness, and self-efficacy was developed to increase screening rates for the most vulnerable groups in five territories of Ain. Table 1 presents the sociodemographic information of each of the five study sample territories compared to Metropolitan France. The target audiences for these programmes were low-income and low-literate women and men aged 50–74 years for colorectal cancer screening, women aged 50–74 years for mammography screening, and women aged 25–65 years for cervical screening. However, very few men ultimately enrolled in the intervention (*n* = 11) and thus, the focus of the analysis comprises the female participants only (*n* = 164).

**TABLE 1:** Sociodemographic information by study sample territories compared to metropolitan France

—	Haut-Bugey Agglomeration^a^	Miribel and the Plateau^b^	Plain of Ain^c^	Pays de Gex^d^	Coastal Region in Montluel^e^	Metropolitan France
**Population in 2017** **^f^**	63,236	23,839	77,644	95,070	24,847	64,639 133
Population density (number of inhabitants per km^2^) in 2017	91.8	363.6	109.1	234.8	194.9	118.8
Number of households in 2017	26,714	9,392	32,837	40,350	9,316	28,734 433
**Homeownership** **^g^**						
Total number of dwellings in 2017	31,810	10,137	38,035	48,994	9,998	34,980 732
Percentage of households owning their main residence in 2017, in %	52.2	66.6	63.9	55.9	66.1	57.6
**Income** **^h^**						
Number of tax households in 2017	25,823	9,170	32,687	33,183	9,091	27,409 461
Share of taxable households taxed in 2017, in %	49.4	64.8	54.8	52.5	59.2	52.1
Median of disposable income per consumption unit in 2017, in Euros	19,920	25,310	22,260	34,520	23,220	21,110
Poverty rate in 2017, in %	16.0	6.7	9.8	13.0	7.3	14.5
**Employment—unemployment (per census**)**^f^**						
Total employment in 2017	27,395	12,150	30,332	19,722	10,480	25,826 145
Change in total employment: average annual rate between 2012 and 2017, in %	−0.9	0.3	1.6	1.6	0.4	0.0
Activity rate for 15–64 year olds in 2017	75.1	76.4	78.3	81.3	79.8	74.1
Unemployment rate for 15–64 year olds in 2017	13.7	9.2	10.5	10.0	8.2	13.4

a Haut-Bugey Agglomeration (42 municipalities including Arbent, Bellignat, Nantua, Oyonnax).

b Miribel and the Plateau (6 towns including Miribel).

c PLain of Ain/Plaine de l’Ain (53 municipalities including Ambérieu-en-Bugey and Saint-Rambert-en-Bugey).

d Pays de Gex (27 municipalities including Prevessin Moens).

e Coastal region in Montluel/Côtière à Montluel (9 communes including Montluel).

f Source: Insee, RP2012 and RP2017 main farms in geography as of January 01, 2020.

g Source: Insee, RP2017 main operation in geography as of January 01, 2020.

h Source: Insee-DGFiP-Cnaf-Cnav-Ccmsa, Localized social and fiscal geography file as of January 01, 2020.

Health promoters contacted 14 non-profit social service organisations serving vulnerable populations in the department of Ain. Subsequently, nine of the 14 organisations agreed to participate in the study. Health promoters went to each of these organisations and held study information sessions whereby they described the study and the eligibility criteria, and answered any questions that potential participants had. They also emphasized that participation was voluntary. Following the information session, interested participants enrolled in the study. The cancer education and prevention intervention was guided by the Health Belief Model [24]. The model suggests that a person’s belief in a personal threat of an illness together with a person’s belief in the effectiveness of the recommended health behavior will predict the likelihood the person will adopt the behavior [24].

The intervention consisted of eight 2-h sessions and was tailored for a low literacy audience (Table 2). The overall objectives of these sessions were to: 1) increase the level of knowledge on health, cancer, and cancer screening; 2) correct misrepresentations about cancer; 3) increase self-efficacy related to cancer screening; and 4) increase cancer screening uptake. Ethical approval for this study was obtained from the Auvergne-Rhône-Alpes Regional Cancer Screening Coordination Center.

**TABLE 2:** Overview of session topics and objectives for the 8-week cancer educational intervention

Session topic	Objectives
**1. Introduction to the intervention and facilitators**	-Develop trust between facilitators and participants
	-Describe objectives of the intervention
	-Distribute tailored educational materials for population with low literacy
**2. Cancer, health and health professionals**	-Acquire knowledge on health and its determinants
	-Acquire knowledge on cancer, cancer screenings (e.g., body parts related to different types of cancers; relevant screening information)
	-Acquire knowledge on the role of each health specialist and the role of the treating doctor
	-Acquire new individual health-related skills
**3. Cancer-related rIsk factors and perceptions**	-Acquire more information about different types of cancer and their risk factors
	-Explore perceptions related to cancer
	-Answer participant questions and debunk cancer-related myths and misperceptions
**4. Colorectal cancer and screening—part 1**	-Exchange and acquire knowledge related to the colon and colorectal cancer
	-Exchange and acquire knowledge related to colorectal cancer screening and its barriers (guided by the health belief model)
**5. Colorectal cancer and screening—part 2**	-Increase awareness of prevention resources, including colorectal cancer screening resources (where, how)
	-Acquire knowledge on the role of the treating doctor in colorectal cancer screening
	-Assist in cancer screening decision-making and how to interpret colorectal screening invitation letter
	-Increase self-empowerment in health decisions and behaviors, including colorectal screening behavior
	-Acquire new individual health-related skills
**6. Breast cancer and screening**	-Exchange and acquire knowledge related to the breast and breast cancer
	-Exchange and acquire knowledge related to breast cancer screening and its barriers (guided by the health belief model)
	-Acquire knowledge on the role of the treating doctor in breast cancer screening
	-Assist in cancer screening decision-making and how to interpret breast screening invitation letter
	-Increase awareness of breast cancer screening resources (where, how)
**7. Cervical cancer and screening**	-Exchange and acquire knowledge related to the cervix and cervical cancer
	-Exchange and acquire knowledge related to cervical cancer screening and its barriers (guided by the health belief model)
	-Acquire knowledge on the role of the treating doctor in cervical cancer screening
	-Assist in cancer screening decision-making and how to interpret cervical screening invitation letter
	-Increase awareness of cervical cancer screening resources (where, how)
**8. Review of cancer-related information and evaluation**	-Review of cancer-related knowledge and relevant screening information
	-Review of cancer screening resources (where, how)
	-Assess knowledge and awareness of cancer and cancer screenings in general, screening barriers and benefits as well as screening self-efficacy

### Data Collection and Analysis

First, the aims of the study were clarified, and informed oral consent was obtained from all participants. Between January 2019 and March 2020, one month following the intervention, we conducted hour-long semi-structured qualitative interviews in French with each participant. None of the women declined participation in the interview. Some interview questions were translated into Arabic, English, and Spanish to ensure that they were understood by each participant. Interview questions were used to examine cancer-related knowledge and awareness (e.g., What did you learn about cancer? Since participating in the health sessions, what do you know about cancer screenings?), screening barriers (e.g., What deterred you from getting screened?) and benefits (e.g., What are some of the reasons one should get screened?) as well as self-efficacy to obtain a cancer screening (e.g., Do you feel capable of taking the necessary steps to get screened?). The questions did not ask about specific cancer screenings; they were concerned with cancer and cancer screenings in general.

The interviews were held in private spaces in the non-profit organizations where the intervention took place. The interviews were audio-recorded and subsequently transcribed. We adopted an inductive, qualitative-driven research design, grounded in an emic or idiographic approach to research which “concerns itself with the specific and unique richness of a phenomenon” [29]. The first three authors began analyzing the data by documenting salient patterns both within each interview and across interviews. We generated a priori codes and developed a codebook based on the Health Belief Model which we used as an analytic framework. Using a line-by-line coding method, we added emergent codes from the data and modified the codebook as needed during the analytic process. Through an interpretive process, we located patterns in the codes and collapsed codes into themes (i.e., data reduction). The first author then conducted qualitative thematic analysis using ATLAS. ti (V.8.0), to search for meanings at the level of coding and for contextualized understandings within the data at the level of interpreting results [30, 31]. The data were then visually displayed in a table to facilitate the interpretation of themes. Stability and agreement are the most relevant types of reliability for textual data. Following category revision, an interrater reliability of 96 per cent was achieved. To see if this agreement was due to chance, the intercoder reliability was tested using Cohen’s Kappa [32]. The overall Kappa coefficient was 0.97.

## Results

Slightly more than half (55.4%) of the participants were between the ages of 25 and 49. Approximately two-thirds attended primary (31.4%) or secondary school (33.7%) and 18.8% had no schooling. More than half of the participants had a beginner or intermediate level of French language comprehension and oral expression (56.5% and 63.4%, respectively). Approximately half of the participants were from Africa (48.6%), a fifth were from Asia or the Middle East (21.1%), and another fifth were from Europe (21.7%). Only one participant was from Latin America (Table 3).

**TABLE 3:** Sociodemographic characteristics of the sample (*N* = 164)

Gender, *n* (%)	
Female	164 (93.7)
Age (years), *n* (%)	
Less than 25 years old	6 (3.7)
25–49	91 (55.5)
50–74	64 (39.0)
74+	3 (1.8)
Education, *n* (%)	
Primary	52 (31.7)
Secondary	56 (34.2)
Post-secondary	8 (4.9)
Other (e.g., special education programmes)	4 (2.4)
No schooling	30 (18.3)
Missing	14 (8.5)
Level of comprehension in French, *n* (%)	
Beginner	31 (18.9)
Intermediate	64 (39.0)
Advanced	51 (31.1)
Missing	18 (11.0)
Level of oral expression in French, *n* (%)	
Beginner	52 (31.7)
Intermediate	52 (31.7)
Advanced	43 (26.2)
Missing	17 (10.4)
Origins, *n* (%)	
Africa	80 (48.8)
Asia and middle East	37 (22.6)
Europe	32 (19.5)
Latin America	1 (0.6)
Missing	14 (8.5)

**FIGURE 3:**
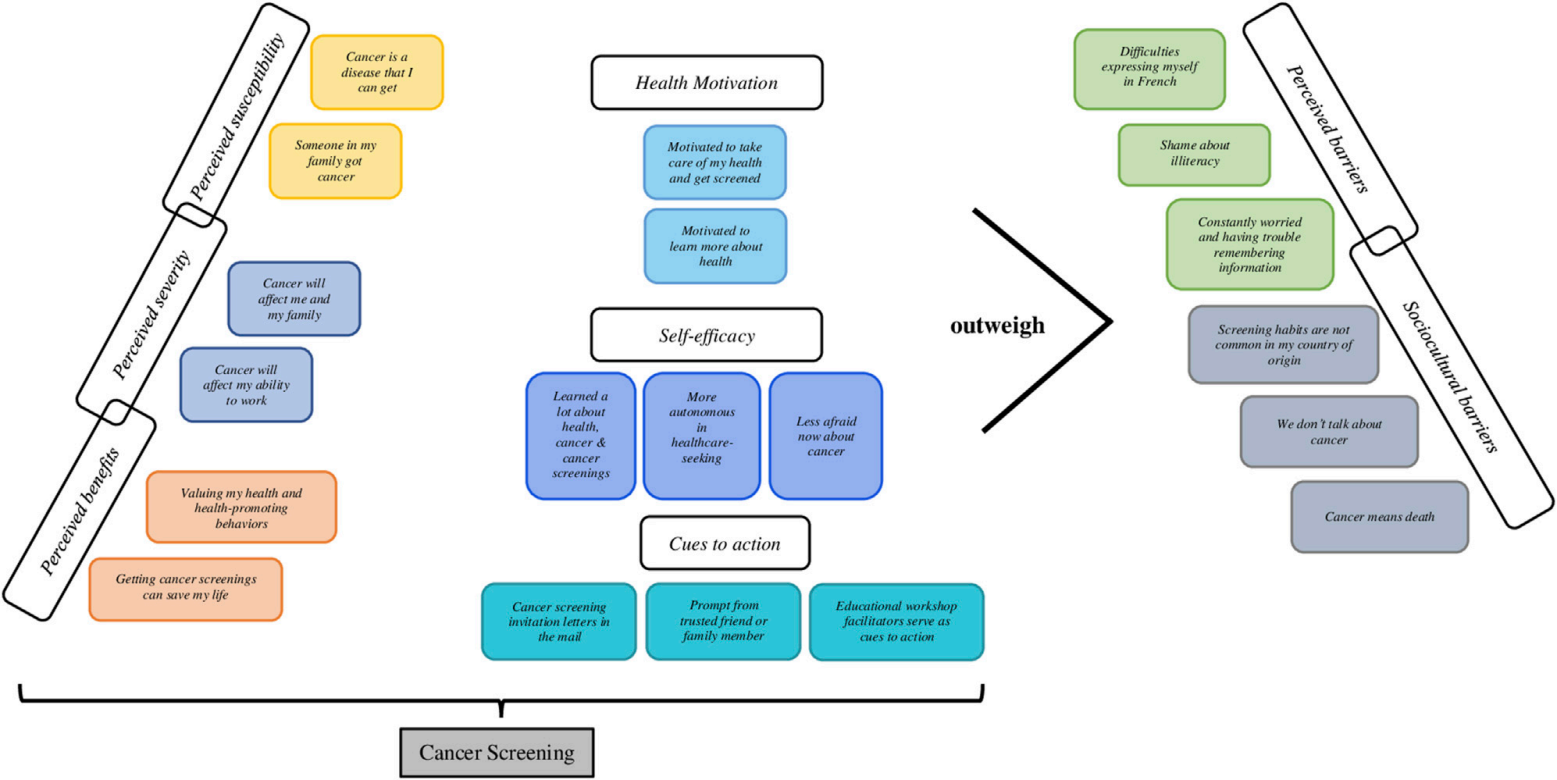
Overview of the salient themes organized using the Health Belief Model as the analytic framework. Action to Promote Organized Cancer Screenings for the Public Developing Literacy in the Ain” (Action de promotion du Dépistage Organisé des Cancers auprès des Publics en cours d'alphabétisation dans l'Ain (ADOCPA), France, 2019–2020. “Action to Promote Organized Cancer Screenings for Populations Developing Literacy in Ain” (Action de promotion du Dépistage Organisé des Cancers auprès des Publics en cours d’alphabétisation dans l’Ain (ADOCPA), France, 2019–2020.

Given that the educational intervention was informed by the Health Belief Model, the salient themes were primarily organized using the constructs of the model (Figure 3). In our analyses, we considered cancer screening in general, given that our questions were not tailored to specific types of screenings. *Perceived susceptibility* refers to beliefs about the probability of obtaining cancer. The HBM predicts that individuals who perceive that they are susceptible to cancer will engage in screening and other preventive behaviors to reduce their risk of developing cancer. Most of the participants (95%) reflected on their subjective assessment of cancer following the cancer prevention educational sessions.

### Cancer Is a Disease That I can Get

*I know now that I too can get cancer. I didn't understand this before (…) Now I know screenings are important. I did my PAP smear. It was normal. I also did a mammogram. There was nothing there either. It was my first PAP smear and mammogram* (Adamaa).

### Someone in My Family got Cancer

*For me, I knew nothing about the disease before. I thought it happened to other people and would not affect me. Then my sister became sick with cancer. I was afraid but after I became more aware of cancer screenings when I did the educational sessions. I think that cancer is a disease that anyone can get, including me, but it can also be cured. It is important to do the cancer screenings* (Wafiya).

*Perceived severity* refers to feelings concerning the seriousness of acquiring cancer and its potential consequences. The HBM proposes that individuals who perceive cancer as serious are more likely to engage in screening and other preventive behaviors. All the participants shared their perspectives on the potential health consequences of cancer (e.g., death, disability, and pain) and its social consequences (e.g., effects on work, domestic life, and social relations).

### Cancer Will Affect me and My Family

*For me, I put on a lot of weight and worried about getting sick if I got cancer. It will affect my ability to take care of my family (…) You see, I'm starting to pay attention, it’s good to protect myself and my health. Before I was always afraid when they sent things home about health (cancer screening invitation letters). I didn’t do it ‘cuz I was always afraid. Now I do the screenings* (Aisha).

### Cancer Will Affect My Ability to Work

*The day we all talked about cervical cancer, I remember the image that I saw, how the tumor grew and it scared me. It is a small task to get a PAP smear and it’s better to discover it when it’s smaller and not to wait until it's too late. I will not be able to work and I will not be able to help my children and family if I get cancer and it is discovered too late. When it’s too late, well it’s too late, I think that all of us who have done these educational sessions, not only me, we all understood the risk that we take if we wait and if we neglect to get screened* (Suhayla).

*Perceived benefits* focus on the individual’s assessment of the efficacy of a screening to reduce the threat of cancer. If an individual believes that screening will decrease its seriousness, then they are likely to engage in the behavior. After the educational sessions, most of the participants (97%) began to realize the value of engaging in health-promoting behaviors to decrease the risk of cancer.

### Valuing My Health and Health-Promoting Behaviors

*We were just working and we didn’t pay attention to our bodies. We never looked at our bodies like that (...) Now we realize that it is important to check the whole body so that we stay healthy* (Hamia).

*It is important for me to* chec*k my whole body. My health is important. I need to check to see if I have something. For example, when I do my bowel movements, I look to see if there’s any blood. I take care of my health. I learned about cancer and prevention screenings I didn't know about* (Isaura).

### Getting Cancer Screenings can Save My Life

Getting a mammogram is important. Now I say ‘I will do it’ so that I can protect myself. If I don’t prevent now, afterwards the cancer will be big and it will become even more serious.

I learned a lot in the educational workshops. Screenings are important because they can detect if there is a problem early on and save your life. I want to have my health because health is life.

*Perceived barriers* refer to the women’s assessment of the obstacles to engaging in health-promoting behavior. The perceived benefits must outweigh the perceived barriers in order for screening uptake to occur. Many of the participants (96%) described a range of barriers.

### Difficulties Expressing Myself in French

All participants shared that language proficiency in French was an obstacle to explaining what they knew about cancer and cancer prevention, to asking further questions, and to expressing themselves openly with health care providers.

*I can't say what’s on my mind, but I understand a lot of things. I don’t speak French. I can’t express myself well to the doctor* (Lulu).

*French, for me, is hard to speak. It is too hard for me to explain myself in French. When someone, like the doctor speaks to me, I understand but I can’t speak. It all stays in my head* (Maelys).

### Shame About Illiteracy

Most of the participants (94%) mentioned how they felt ashamed about their illiteracy in the clinical encounter.

*I cannot read or write (…) I feel ashamed about myself, even in front of the doctor* (Salima).

*I didn’t dare to talk to my doctor. I feel dumb because I can’t read or write well. In France, I’m too stressed at the doctor’s office* (Yasmeen).

### Constantly Worried and Having Trouble Remembering Information

Many participants (96%) also expressed that they had a lot on their minds that preoccupied them. Thus, it made it difficult for them to retain information sometimes. These worries were related to their precarious living conditions and affected their ability to learn effectively.

*Yes, the information is gone out of my head. I forget it. My head spins with worry. My kids have problems at school. My husband cannot find a job and money is tight. His French is not good enough either* (Amina).

*There’s no one to help me, I’m the one who takes care of everything. When I finish the educational workshops, I’m very tired. I forget, I have too much to think about. When you have problems, it’s hard to remember things. I have too much stress* (Habiba).

### Sociocultural Barriers

Although culture-specific barriers are not constructs in the Health Belief Model, these barriers were salient in the data analysis. *Sociocultural barriers* refer to specific shared values and beliefs of a self-identified ethnic/cultural group that may impede participation in a cancer screening.

### Screening Habits Are not Common in My Country of Origin

Many (92%) participants mentioned that cancer screening was not common in their country of origin. They described not having a “habit” of going to the doctor proactively to get screened. These women also mentioned that unless they felt symptoms, they were not likely to go to the doctor.

*Screening is not what we normally do in our country of origin (…) We are not used to it. We do not go to the doctor unless we feel something bad* (Cyra).

### We don’t Talk About Cancer

Many women (97%) stated that they avoid talking about cancer with other women in their families and communities. It is a topic that they find very difficult to bring up with others.

*We don’t talk about it at all, it’s not a subject to talk about. But after we attended, we understand, we discover. Already people say ‘this disease, may Allah preserve us from it’ [says this in Arabic and then resumes in French]. We do not even pronounce the word ‘cancer.’* (Farrah).

*With my family in Kosovo, we never talked about cancer. In Kosovo, it is not common to talk about cancer. It is complicated to talk about cancer with others, the people would rather run away than talk about it* (Aferdita).

### Cancer Means Death

Nearly all the participants (98%) also mentioned that cancer is typically associated with death.

*You need the courage to talk about it (…) for us, cancer means death* (Aliz).

*Because we think that when we say cancer or when someone has cancer, that’s it, that person is dead* (Misha).

*Health motivation* has also been used as part of the HBM in predicting health-related behavior. It refers to a generalised state of intent that results in the uptake of health-promoting behaviors.

### Motivated to Take Care of My Health and Get Screened

Despite their perceived barriers, all the participants shared that their increased level of awareness following the educational sessions motivated them to be more health-conscious and obtain their cancer screenings.

*I am more motivated now. I learned a lot with you in the educational sessions. I want to take care of myself. My health is very important. I also know more about cancer screenings and the cancer stages. It is important not to neglect our health and do the screenings* (Haniya).

### Motivated to Learn More About Health

All of the participants also shared that they wanted to obtain more health education and were motivated to continue to learn more about their health going forward. They all stated that they needed and wanted more sessions so that they could learn more about cancer, other diseases, and preventive health services.

*It would be good if we could learn more about health. I would like to participate in more educational sessions* (Nawar).

*It’s not good to stop learning about our health. I’d like it if you could come back next year so we can understand more about cancer and prevention* (Zubeida).

*Cues to action:* The HBM posits that a cue, or trigger, is necessary for prompting engagement in health-promoting behaviors. Cues to action can be internal or external. Physiological cues (e.g., pain, symptoms) are an example of internal cues to action. External cues include events or information from sources that promote engagement in health-related behaviors.

### Cancer Screening Invitation Letters in the Mail

Many participants (98%) acknowledged receiving cancer screening invitation letters in the mail. However, many of them (90%) stated that they did not understand that these letters were recommending a specific cancer screening for which they were eligible and ended up throwing out the letters. Following the educational intervention, they all stated that they now understood how to recognize these invitation letters.

*I did not know what the letter was or what it was recommending. Because I did not understand it, I threw it in the trash. Now I know what they are for* (Marjani).

*I thought the letter was an advertisement and I threw it out. Now I learned that the letter is to inform me about getting a specific cancer screening and do not throw it out* (Zohra).

### Prompt From Trusted Friend or Family Member

Many participants (96%) expressed how getting information and encouragement from a trusted friend or family member about getting screened prompted them to do so.

*I shared what I learned in the educational sessions with my husband. My husband told me that it was a good thing for me to get tested. He said, ‘Go and get the screening.’* So I did (Kaamla).

*When I discovered that I had a lump in my breast, my sister was the one who told me, ‘I will go with you to get tested. You are not alone.’ This gave me the courage to get a mammogram* (Malika).

### Educational Workshop Facilitators Serve as Cue to Action

Many of the participants (97%) stated that the educational facilitators served as a key cue to action for them to schedule a cancer screening. Nearly all the participants (99%) stated that they trusted the facilitators, felt very comfortable sharing their worries with them, and learned information related to health, cancer, and cancer prevention from them. All of the participants stated that they enjoyed learning from the facilitators as well.

*You talk like a sister or a mother. I was afraid to ask the doctor questions, but with you, it’s okay. I'm happy with you. I learned about the different cancer screenings. Now I will be less afraid with the doctor* (Zoubeida).

*I like learning from you [facilitator] and talking to you (...) people who give us their time and who can be trusted. And you explain everything so well. I learned a lot from you. I am more aware about cancer and what to do now compared to before. I will get an appointment for my mammogram* (Amane).

*Self-efficacy* refers to the participant’s perception of her ability to successfully perform a health-promoting behavior such as obtain a cancer screening. Confidence in one’s ability to affect a change in outcomes is a key component of health behavior change.

Learned a lot about health, cancer, and cancer screenings.

All of the participants described how in the educational workshops, they learned a lot about health in general, and about cancer and cancer prevention, specifically.

*When I accompany my husband who has epilepsy to the hospital, I can now read the sign for ‘neurology department’ and I know where to go. I learned with you. I learned a lot of things I didn’t even know, not only about cancer and screenings, but about health as a whole* (Saeeda).

*I understood a lot of things about health thanks to you. For a long time, I didn't understand. I am very happy to have learned a lot about health with you. I feel that I can get a mammogram now* (Laila).

### More Autonomous in Healthcare-seeking

Beyond increasing awareness related to cancer and cancer prevention, most of the participants (95%) also stated that they became more autonomous with issues related to their own health and with those of their family members after they participated in the educational workshops. Many women (93%) shared that they felt more capable of expressing themselves to the doctor following the educational sessions.

*I am proud of myself. Before I didn't know anything at all but now it’s okay, I can go alone now to the doctor’s office and I am not afraid* (Imane).

*Before when I went to the doctor, I was always accompanied by someone. But now I can go alone to get a mammogram* (Bayo).

*I’m a good listener. I like to learn things about health that I don’t know. I try to understand things. I like to know about my body* (Fatima).

*I have more courage now to talk to the doctor. I don’t have the fear and the shame like before. Now it’s easier to talk to the doctor and explain things* (Majeeda).

*It’s good how you taught us about cancer and screenings, and how to make an appointment with the doctor. Now I go by myself [...] I’m going to the pharmacy too by myself. I learned things I didn’t know. Now I have a little bit more experience, and less anxiety than before. I’m learning new health words. I can use what I learned to talk to my doctor* (Farah).

### Less Afraid now About Cancer

Many women (94%) also expressed that as they learned more about cancer and cancer prevention during the educational workshops, they felt less afraid and more capable of talking about cancer.

*At first, I was afraid of cancer, but after talking about it, I’m less afraid. With the information we received, I am less scared. I am less stressed about getting screened. I know what to do. I can get tested* (Naima).

*I was scared especially for the PAP smear. Now I’m not scared anymore. The educational sessions helped me. For example, I went to do a PAP smear and this test was good to remove my worries* (Samira).

## Discussion

Efforts to engage disadvantaged populations in cancer prevention initiatives will be hindered if there is limited understanding of their knowledge, awareness, self-efficacy, and perceptions of cancer and cancer screenings. This study, therefore, makes an important contribution to the global public health literature. To our knowledge, it is the first qualitative study of its kind that provides such evidence among low-income, illiterate immigrant women in France.

Findings revealed that following the cancer education and prevention intervention, the participants expressed having gained more cancer and cancer prevention knowledge and awareness. Specifically, they evolved in their understanding of cancer as a disease to which they too are susceptible. They also realized the potential negative health consequences of being diagnosed with cancer such as their inability to work and/or take care of their families. As predicted by the Health Belief Model, the participants who perceived that they were susceptible to cancer and realized the seriousness of the disease and its consequences, expressed more motivation to get screened [24]. Similar to our study’s findings, Bridou et al.‘s study [33] revealed that the main psychological facilitators of colorectal screening included having information about colorectal cancer screening and individuals’ perception of risk to getting colorectal cancer. Moreover, a systematic review conducted by Honein-AbouHaidar et al. [34], demonstrated that the decision to participate in colorectal cancer screening depended on an individual’s awareness of colorectal cancer screening; increased awareness contributed to higher motivation for screening.

The literature also points to inadequate language and health literacy skills as key drivers of suboptimal screening [19–22, 34–39]. Consistent with the literature [19–22, 35], all the female participants stated that limited French proficiency was a main barrier to seeking more health information, expressing their concerns, demonstrating their acquired health knowledge, and getting screened. For example, Francois et al.‘s study [35] also described how many Haitian immigrant patients in New York City felt that their lack of proficiency with the English language in was a significant barrier to health care access.

Our study findings also provide a more nuanced picture in that the immigrant women participants shared that they felt shame about their illiteracy in general, including in the patient-doctor encounter. They also experienced constant worry as they navigated their daily challenges, which also created barriers to their learning and retention of health information. These women’s precarious environments characterized by economic insecurity and family stress may impede their ability to prioritize cancer screenings and their health, more generally [14–17, 40].

Participating in the cancer education and prevention intervention served as a prompt for these women to learn more about health, cancer, and cancer screenings as well as value health-promoting behaviors. The intervention also created an opportunity for them to decrease their fear about cancer and become more autonomous in seeking preventive healthcare services. As per the HBM, the perceived benefits outweighed their perceived barriers to obtaining a screening [24]. These findings are consistent with the findings of other French studies [33, 39].

### Limitations and Strengths

Several study limitations must be noted. Given that our study used a purposive sample, the findings are not generalizable beyond the study sample. Additionally, slightly more than half of the participants were between the ages of 25 and 49 and were, therefore, not directly concerned by breast and colorectal cancer screening. We also did not examine specific cancer-related knowledge, awareness, self-efficacy, and perceptions of barriers for each cancer screening (colorectal, breast, and cervical). Instead, we asked questions about cancer in general that cut across different types of cancer and cancer screenings. We were not able to obtain information regarding the determinants specific to each screening. We also were not able to include men’s perceptions in our analyses given the low number of men who enrolled in the study. Further research is recommended to elucidate the specific facilitators and barriers for each type of screening as well as to examine any gender differences.

Despite the limited generalizability, we conducted a rigorous, qualitative thematic analysis, which facilitated the identification of more nuanced themes that have theoretical and practical relevance, and are not apparent from data collected using structured surveys and quantitative research methods. The study was innovative and contributed to extant literature in that it generated new and in-depth knowledge related to low-income and illiterate immigrant women’s cancer-related knowledge, awareness, self-efficacy, and perceived screening barriers. Other strengths of this study include the use of a community-based research approach, a sample size with a substantive number of participants, the development of a pilot intervention on a smaller scale prior to implementing the full-scale intervention for its effectiveness, and the use of multiple coders to check reliability of results.

### Implications for Practice and Policy

The study findings have implications for cancer prevention education and practice. Specifically, the participants expressed several key cues to getting screened, including receiving cancer screening invitation letters in the mail and a prompt from a trusted health messenger. These findings corroborate those of other studies [41–44]*.* For example, in another French qualitative study, Le Bonniec et al. also demonstrated that being encouraged to get screened served as an important screening facilitator [39]. A health provider who is sensitive to the patients’ concerns can serve as an important cue to action for disadvantaged populations [41, 42]. It is important that the health provider convey understanding, care, and respect to these illiterate patients in a way that fosters mutual trust and builds the patients’ self-confidence and self-efficacy to ask questions and share concerns [42, 45].

The study findings also have implications for healthcare practice and policy. Specifically, the findings inform our understanding of patient-centered and culturally competent health education and healthcare with underserved and illiterate immigrant populations. Firstly, they provide useful information for health clinicians and highlight the importance of increasing their cultural awareness and competence to foster productive patient-centered interactions and to help promote screening among members of these disadvantaged communities. Tailoring health education and cancer prevention interventions is a central tenant of patient-centered care and has been shown to be associated with increased cancer screening adherence, engagement in care, and improved outcomes of care [23, 46].

Secondly, these findings provide useful data for administrators and policymakers in the cancer prevention arena to develop more culturally appropriate and effective audience-centered models and policies related to service delivery across the cancer continuum [23]. Thirdly, study findings shed light on the importance of developing effective targeted health education and cancer prevention communication strategies and materials. Given the low literacy level among these populations, cancer and cancer prevention materials need to be adapted to ensure that the target audience can comprehend the information.

Health care organisations often remind patients of screenings *via* letters sent in the mail. Such reminders can increase screening rates, but only if the messages are understood by the recipients, as our findings reveal. Indeed, previous studies demonstrate how audience-centered approaches matter when developing materials and intervention strategies focused on addressing health behaviors such as the uptake of cancer screenings [23, 47]. Furthermore, given that prompting methods such as cancer screening invitation letters in the mail are often difficult to sustain, interactive voice response and other mobile health (mHealth) methods may hold promise as a feasible, cost-effective strategy to promote cancer screening uptake.

### Conclusion

The study findings suggest that addressing cancer prevention requires distinct approaches rather than a one-size-fits-all approach, taking into account the differences in levels of literacy, income, health knowledge, awareness, self-efficacy, and perceived barriers. Efforts to effectively promote cancer screening will be critical for achieving the goals of the Ministry of Health for the Auvergne-Rhône-Alpes region, the French National Cancer Institute, and Santé Publique France, the national public health government agency, to increase overall screening rates and reduce cancer and cancer screening inequities that disproportionately impact vulnerable populations.
